# A machine learning approach for modeling the occurrence of the major intermediate hosts for schistosomiasis in East Africa

**DOI:** 10.1038/s41598-024-54699-1

**Published:** 2024-02-21

**Authors:** Zadoki Tabo, Lutz Breuer, Codalli Fabia, Gorata Samuel, Christian Albrecht

**Affiliations:** 1 Department of Animal Ecology and Systematics, Justus Liebig University Giessen, Heinrich-Buff-Ring 26 (iFZ), 35392 Giessen, Germany; 2 Institute for Landscape Ecology and Resource Management, Justus Liebig University Giessen, Heinrich-Buff-Ring 26 (iFZ), 35392 Giessen, Germany; 3 Centre for International Development and Environmental Research (ZEU), Justus Liebig University Giessen, Senckenbergstrasse 3, 35390 Giessen, Germany; 4 Department of Environmental Science, Faculty of Science, University of Botswana, P/Bag UB00704, Gaborone, Botswana

**Keywords:** Freshwater snails, Schistosomiasis, Predictor features, Random forest, Sub-Saharan Africa, Ecology, Environmental sciences

## Abstract

Schistosomiasis, a prevalent water-borne disease second only to malaria, significantly impacts impoverished rural communities, primarily in Sub-Saharan Africa where over 90% of the severely affected population resides. The disease, majorly caused by *Schistosoma mansoni* and *S. haematobium* parasites, relies on freshwater snails, specifically *Biomphalaria* and *Bulinus* species, as crucial intermediate host (IH) snails. Targeted snail control is advisable, however, there is still limited knowledge about the community structure of the two genera especially in East Africa. Utilizing a machine learning approach, we employed random forest to identify key features influencing the distribution of both IH snails in this region. Our results reveal geography and climate as primary factors for *Biomphalaria*, while *Bulinus* occurrence is additionally influenced by soil clay content and nitrogen concentration. Favorable climate conditions indicate a high prevalence of IHs in East Africa, while the intricate connection with geography might signify either dispersal limitations or environmental filtering. Predicted probabilities demonstrate non-linear patterns, with *Bulinus* being more likely to occur than *Biomphalaria* in the region. This study provides foundational framework insights for targeted schistosomiasis prevention and control strategies in the region, assisting health workers and policymakers in their efforts.

## Introduction

A large number of neglected tropical diseases (NTD) in sub-Saharan Africa account for approximately 200,000 deaths annually as well as 57 million lost life-years^1^.The most significant of these diseases, schistosomiasis, is the second most prevalent parasitic disease only after malaria in several sub-Saharan African countries^1,2^, severely affecting low-income rural communities with poor sanitation^3^. Schistosomiasis negatively impacts child development, pregnancy outcomes, and agricultural productivity, perpetuating poverty for millions of Africans^1,3,4^. In spite of only making up 13% of the global population, sub-Saharan Africa accounted for 90% of schistosomiasis cases^5^.

Human schistosomiasis is caused by species of schistosome trematode worms: *Schistosoma mansoni*, *S. haematobium*, *S. japonicum*, *S. intercalatum*, and *S. mekongi*. These infections manifest in two main forms: intestinal schistosomiasis, attributed to *S. haematobium*, and urogenital schistosomiasis, associated with other species such as *S. mansoni*^6,7^. The life cycle of *Schistosoma* initiates when parasitic eggs from infected human feces or urine enter freshwater sources. Under favorable environmental conditions, these eggs hatch into miracidia, which actively seek out and penetrate suitable IH snails. Asexual reproduction occurs within the snails, leading to the development of cercariae. At this advanced stage, the cercariae are released into the water as free-living parasites and can penetrate human skin, thereby completing the cycle and causing the disease^6^. Notably, *Bulinus* and *Biomphalaria* snails act as IHs for S*. haematobium* and *S. mansoni*, respectively^7^. *Schistosoma haematobium* and *S. mansoni* are prevalent in Sub-Saharan Africa, significantly contributing to the burden of schistosomiasis.

While the 2020 goal for schistosomiasis elimination proved elusive^8^, control efforts in Sub-Saharan Africa, specifically East Africa, have predominantly relied on mass chemotherapy, particularly for school-aged children^9,10^. However, recognizing the inadequacy of this approach alone, there is an urgent call for alternative strategies^10^. The integration of One Health into the WHO 2030 NTD roadmap, encompassing human treatment, livestock treatment and/or vaccination, environmental management, and snail control, has garnered increased recognition for its potential impact^11,12^. From an ecosystem perspective, factors influencing the presences of *Schistosoma* parasites and their snail hosts can significantly impact the transmission dynamics of schistosomiasis^12^. Investigating such factors aligns with the WHO 2030 NTD elimination strategy^11^. Nonetheless, targeting IH snails, demonstrated as effective^13^, holds promise. However, our understanding of various aspects related to snail hosts remains limited, with a scarcity of studies providing comprehensive prevalence data and identifying significant features influencing the distribution of *Biomphalaria* and/or *Bulinus* IH snails^14,15^. This knowledge gap is particularly pronounced in East Africa overall, with persistent schistosomiasis hotspots in Kenya and Tanzania^9^.

Machine learning techniques, particularly random forest (RF)^16^, have gained wide application in various scientific domains for classification and regression analyses pertaining identification of significant features^14,17–20^. In classification tasks, RF has demonstrated superior predictive accuracy compared to other methods, such as logistic regression^21–23^. RF is resilient to multicollinearity, a common issue in ecological datasets^24^. It also offers effective solutions for addressing missing data^25^. RF aids in discerning predictor variables with substantial influence on response variables, distinguishing them from those that may not contribute significantly. Therefore, this research aims to provide comprehensive insights into the distribution of IHs in the East African region using RF to identify the spatial distribution of IH snail distribution and the significant features driving their distribution.

Currently, only one documented study exists for East Africa region as a whole, albeit restricted to *Biomphalaria* IHs and a limited number of surveyed locations, considering just eight predictor features^26^. This previous study gives a first impression, however, obtaining robust results may necessitate the inclusion of a broader array of potential features in the analysis. This challenge becomes especially complex in regions like East Africa characterized by variable occurrences of both *Biomphalaria* and *Bulinus* IH species, in conjunction with diverse geographical, climatic, environmental, and anthropogenic factors. This highlights a substantial gap in understanding the distribution of the two genera, which are the primary contributors to the schistosomiasis burden in the region. To address this knowledge gap, our study has two primary objectives: a) to assess the significance of a broader array of potential features, including climatic, environmental, topographic, and human impact factors, in influencing the distribution of IH snails of both *Bulinus* and *Biomphalaria* snails in East Africa, and b) to determine the anticipated probability of occurrences for the pertinent species within the genera based on the most significant factors.

## Material and methods

### Description of study area

The study area spans the East African region, including Uganda, Kenya, and Tanzania, situated within the Tropics of Cancer and Capricorn. East Africa covers an extensive area of approximately 6667 Mio ${{\text{km}}}^{2}$ and is home to roughly 488 million people, making it the most densely populated sub-region in Africa^27^. This region is rich in freshwater sources, such as swamps, rivers, and (crater) lakes, but also man-made structures such as dams and irrigation schemes, serving as potential habitats for IH snails^14,28,29^. In addition, East Africa exhibits a diverse range of geographical, climatic, hydrological, and human-induced factors, all of which are highly relevant for the distribution of IH snails. Importantly, both *S. mansoni* and *S. haematobium* are major disease burdens in the region associated with the presence of both *Bulinus* and *Biomphalaria* species^14,30^.

### Occurrence and geographic data

**Figure 1:**
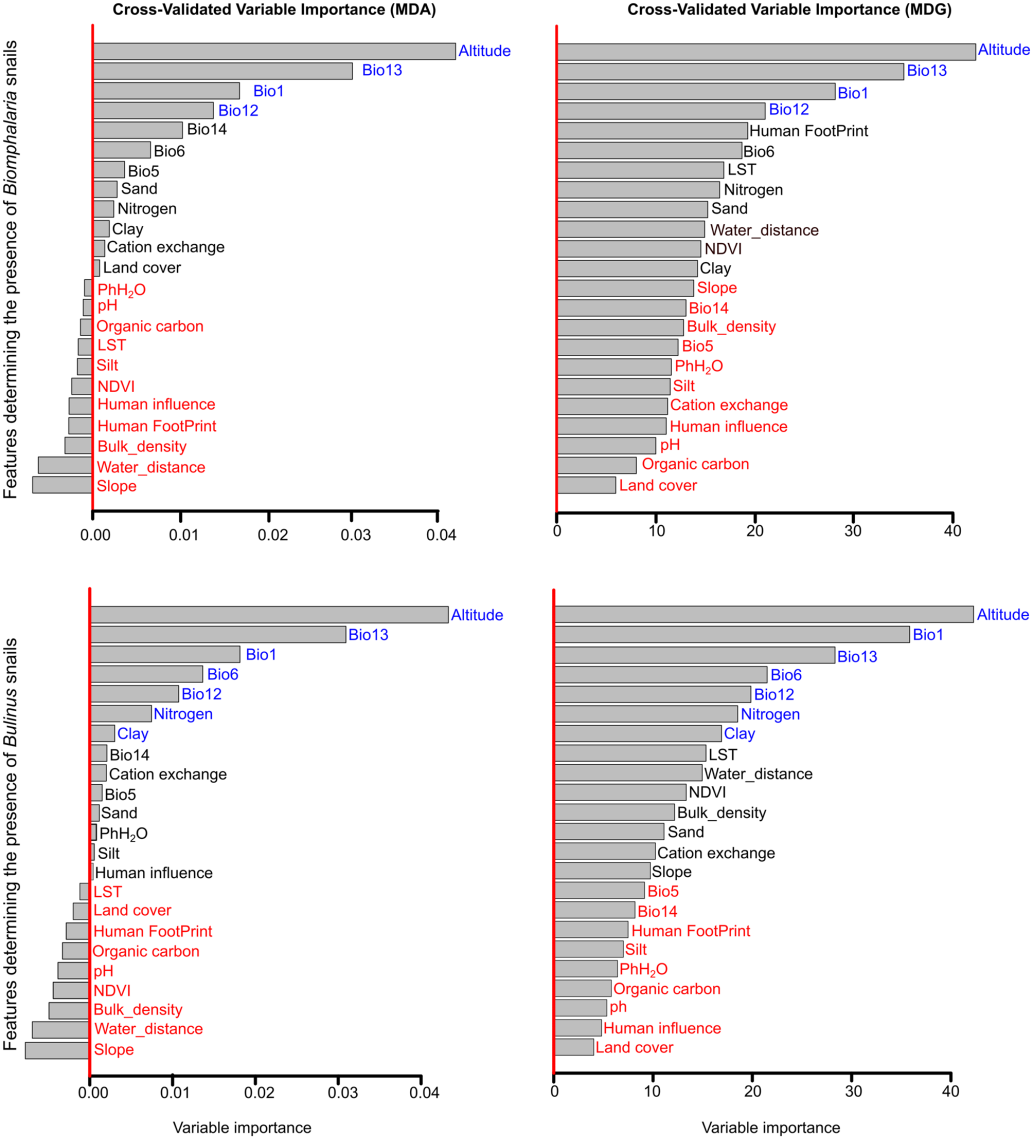
Contributions of the predictor features to the distribution of *Biomphalaria* (upper panel) and *Bulinus* (lower panel) considering the variable importance by mean decrease in accuracy (MDA, left)) and mean decrease in Gini (MDG, right). The prominently significant features are highlighted in blue, those with minor influence are marked in black, and those in red are considered non-significant. For abbreviations of features see Table 1.

The geographic distribution of occurrence data for the *Biomphalaria* and *Bulinus* IH snails in the study area can be found in the Supplementary File S1 Fig. 1. We collected geographic data (longitude and latitude), pertaining to *Bulinus* and *Biomphalaria* distribution in the three East African countries Uganda, Tanzania and Kenya, including data previously reported by Chibwana et al.^31^, Tumwebaze et al.^32^, Tabo et al.^14^, as well as those reported in the Global Biodiversity Information Facility (GBIF), that include recent data from the museum specimens and DNA barcodes^33^. The information obtained from GBIF constitutes secondary data retrieved online, whereas the remaining three sources involve primary data collected through field surveys. This dataset encompassed all *Biomphalaria* species, universally acknowledged as hosts, and selectively featured specific well-documented host species of *Bulinus* (see the Supplementary Table S1). After obtaining the data, we imported it into the R statistical environment, version 4.0.3^34^, and conducted a thorough data cleansing process by removing duplicate records. Subsequently, we harnessed the processed geographic data to extract environmental, climatic, topographic, soil content, and human influence drivers associated with occurrence data of IHs using the R programming language, Google earth engine^35^, and the ArcGIS Pro geographical information systems (GIS), as briefly described in Sects. "Climatic and environmental features"–"Human impact features".

### Climatic and environmental features

Climate factors such as temperature, precipitation, and natural habitat conditions are recognized for their impact on host snail distribution patterns^36–38^. To account for the potential preference of IH snail species for climatic variations, we obtained high-resolution bioclimatic data from the WorldClim (v2.1) global dataset, typically spanning records from 1970 to 2000 with a spatial resolution of 340 km^2^ (10-arc minutes)^39^, within the R statistical environment. We excluded most bioclimatic features and selected mean annual temperature (BIO1), temperature of the warmest month (BIO5), temperature of the coldest month (BIO6), annual precipitation (BIO12), precipitation of the wettest month (BIO13), and precipitation of the driest month (BIO14), which have been extensively documented for their impact and the biological relevance for the presence and distribution of IH snails^14,37,38^. In addition, we computed the mean land surface temperature (LST) using the MOD11A1.061 Terra Land Surface Temperature and Emissivity Daily Global 1 km dataset within Google Earth Engine , an indicator of energy exchange at the land surface-atmosphere interface known for its influence on climate and ecosystems^40^. We have averaged all LST data for the years 2000, 2010, and 2020, accommodating any temperature and emissivity fluctuations over the past two decades. In the Google Earth Engine platform, we scripted the extraction of the Normalized Difference Vegetation Index (NDVI) from the MODIS product MOD13Q1 (2021) V6.1, offering valuable information at a 250 m pixel resolution^41^. We have averaged NDVI data for the years 2000, 2010, and 2020, accounting for any fluctuations in the index over the past two decades. The NDVI is a widely-used indicator for the quantification of vegetation health and density^42,43^.

In addition, land cover, which is known to significantly impact snail habitat suitability^44^, was considered and extracted from the MODIS Land Cover Type Yearly Global 500 m dataset via Google Earth Engine^45^. The land cover classification employed in this study distinguishes 17 land cover classes, including 11 natural vegetation classes (such as forests, open herbaceous areas, and wetlands), 3 human-altered classes (comprising agricultural land and built-up areas), and 3 non-vegetated classes (including snow, rocks, and water bodies). Furthermore, various physiochemical properties previously studied for their effects on IH snail distribution^14,26,46^ were integrated into our analysis. This included soil pH, soil organic carbon content in fine earth, and soil cation exchange capacity obtained at a 30 m resolution at a depth of 0–20 cm and 20–50 cm from the Innovative Solutions for Decision Agriculture Ltd (iSDA) data set via Google Earth Engine^47^. Additionally, data on soil composition, including clay, sand, silt, nitrogen content, and pH (measured in H_2_O) at a depth of 0-5 cm, were sourced from the International Soil Reference and Information Centre (ISRIC), the World Soil Information Service^48^.

### Topographic features

We included topographic metrics, such as altitude, slope, and distance to the next water body as surrogate indicators of biogeographical isolation, which can influence colonization and limit dispersal, potentially impacting IH establishment in the region^14,49^. Altitude data, a key topographic factor affecting snail host distributions and prevalence of schistosomiasis^50^, was obtained from the WorldClim database. Slope was derived from the Shuttle Radar Topography Mission (SRTM) digital elevation data using Google Earth Engine at approximately 30 m resolution^51^. The nearest distance from occurrence points to surface water bodies was calculated using the "Near" tool in ArcGIS^52^.

### Human impact features

We integrated two significant indices, the Human Influence Index (HII) and the Human Footprint Index (HFI), to assess the impact of human activities on the distribution of IH snails. We obtained HII Data from the Last of the Wild Project (version 2, 2005) at a spatial resolution of 1 km from NASA's Socioeconomic Data and Applications Center (SEDAC). This dataset quantifies relative human impact within each terrestrial biome using scores, derived from 9 global data layers. These layers include factors such as human population pressure (population density), human land use and infrastructure (built-up areas, nighttime lights, land use/land cover), and human access (coastlines, roads, railroads, navigable rivers)^53^. Scores range from 0 to a maximum of 72, with higher scores indicating greater human influence and lower scores suggesting less human influence. Likewise, we acquired HFI data from the Last of the Wild Project (version 3, 2009) through SEDAC (NASA) with a spatial resolution of 1 km. The dataset encompasses eight variables, such as built-up environments, population density, electric power infrastructure, crop lands, pasture lands, roads, railways, and navigable waterways. Scores within the range of 0 to 50 were assigned, where higher scores signify increased human influence and lower scores indicate less human influence^53^. We acquired region-specific data for both HII and HFI in a geographic coordinate system (GCS) from the SEDAC webpage, then extracted pixel-level data for both indices using the "Extract Values to Points" tool in ArcGIS. Note that SEDAC was preferred because it provided the most recent spatial/geographic data for both HII & HFI.

### Data analysis

For assessing the importance of predictor features in both *Bulinus* and *Biomphalaria* RF models, we applied a cross-validation based on presence or absence (1/0) feature sensitivity, a widely-used resampling technique to evaluate generalization capabilities and prevent overfitting^54^. Cross-validation serves to evaluate the stability of variable rankings and mitigates the influence of randomness in the assessment process. The significance of individual parameters in the overall RF models was evaluated using two crucial metrics, Mean Decrease in Accuracy (MDA) and Mean Decrease in Gini (MDG)^17^. MDA is suitable when the goal is to maximize the overall accuracy of the classification model while MDG is often used when the goal is to build decision trees that create nodes with high homogeneity, resulting in better separation of classes^17^. Notably, variations in MDA and MDG outputs are common due to distinct calculation approaches and metrics. Addressing ranking disparities between MDA and MDG, we incorporated both metrics but primarily underscored features deemed significant by both metrics. Thus, when interpreting variable importance, it is advisable to prioritize relative rankings over comparing absolute values between these two measures, ensuring a more comprehensive understanding of feature significance and analytical robustness. In addition, to visually represent how individual predictor features influence the behavior of each IH snail in the region, we employed partial dependence plots^14,55^. The plots illustrate the relationship between a specific significant variable and the occurrence of the species while keeping all other variables constant.

## Results

### Occurrences of IH snails and associated predictor features

The data consists of a total of 455 recorded occurrences for *Bulinus* (52%) responsible for the transmission of *S. haematobium*, and 412 (48%) for *Biomphalaria* transmitting *S. mansoni*. Specifically, the dataset encompassed 77, 69, and 309 records for *Bulinus* species and 134, 143, and 135 records for *Biomphalaria* species in Uganda, Kenya, and Tanzania, respectively.

Overall, we considered 23 predictor features for the RF model. Their spatial resolution, potential mean value, standard deviation, and range variation are shown for both genera (Table 1). Detailed occurrence data, along with corresponding geographic information for the IH snails, can be found in the Supplementary Table S1. In general, both genera share parameter values that exhibit minimal spatial variation considering range of their potential predictors. This similarity in most features is potentially influenced by the location of the region within the same tropical climate zone favoring both species. For example, the data shows that the altitudinal range for *Biomphalaria* ranges from 46 to 2342 m.a.s.l, while for *Bulinus*, it spans from 3 to 2058 m.a.s.l. Additionally, soil conditions in the region, which tend to be alkaline, reflect a complex interplay of various soil components (clay, silt, sand), soil cation exchange capacity, and the bulk density of the fine earth fraction. The high nitrogen content (0.5–4.6 g kg^–1^) in the area can be attributed to emissions from decomposing organic matter such as vegetation (index range 0.18–0.8), land cover, and human activities like deforestation. Nevertheless, an evaluation of the significance of individual parameters in the cross-validated random forest models for *Biomphalaria* and *Bulinus* has been conducted and is presented in Sect. “Variable importance”.

**Table 1:** The input predictor parameters, their spatial resolution; mean values, standard deviation, and the range: *Buli Bulinus* species, and *Biom Biomphalaria* species.

Parameter	Abbreviation	Spatial resolution	Sources	Mean values		Standard deviation		Range	
				Buli	Biom	Buli	Biom	Buli	Biom
Mean annual temperature (ºC)	BIO1	340 km^2^	WorldClim2	21.6	18.4	74.87	7.2	2–27.5	2–29.1
Temperature of the warmest month (ºC)	BIO5	340 km^2^	WorldClim2	22.0	23.3	13.04	11.0	28–34.3	3–36.6
Temperature of the coldest month (ºC)	BIO6	340 km^2^	WorldClim2	14.5	12.3	6.18	4.55	11–21.3	8–22.1
Mean annual precipitation (mm)	BIO12	340 km^2^	WorldClim2	1168	718	544	604	8–1989	1–1918
Precipitation of the wettest month (mm)	BIO13	340 km^2^	WorldClim2	272	163	119	83	12–522	13–522
Precipitation of the driest month (mm)	BIO14	340 km^2^	WorldClim2	27	30	17.36	21.9	1–72	1–91
Land surface temperature (ºC)	LST	340 km^2^	MODIS	25.3	26.7	6.79	5.4	3.4–36.18	12–43.6
Normalized difference vegetation index	NDVI	250 m^2^	MODIS	0.58	0.59	0.10	0.13	0.18–0.83	0.18–0.8
Land cover	Land cover	500 m	MODIS	8.26	6.9	4.46	5.21	1–14	1–14
Soil pH	pH	30 m	iSDA	5.96	5.9	0.44	0.6	5.1–7.8	5.1–9
Soil organic carbon content in fine earth (g/kg)	Organic carbon	30 m	iSDA	6.76	8.3	2.45	3.3	1.23–17.18	1.23–17.17
Soil cation exchange capacity (cmol( +)/kg)	Cation exchange	30 m	iSDA	10.88	14.6	6.74	6.5	1.23–35.59	1.23–32.12
Bulk density of fine earth fraction (g/cm^3^)	Bulk_density	30 m	iSDA	1.32	1.27	0.09	0.09	1.12–1.57	1.11–1.7
Clay content (%)	Clay	340 km^2^	ISRIC	29.95	36.8	12.1	11.9	2–61	3–61
Sand content (%)	Sand	340 km^2^	ISRIC	38.64	33.7	16.9	13.6	3–72	2–61
Silt content (%)	Silt	340 km^2^	ISRIC	19.3	19.9	6.84	8.8	1–35	2–32
Nitrogen content (g kg^-1^)	Nitrogen	340 km^2^	ISRIC	1.36	1.9	0.51	0.79	0.5–4.6	0.62–4.6
pH in H_2_O	pH_H2O_	340 km^2^	ISRIC	6.05	6.08	0.53	0.63	5.32–8	5.3–9.2
Altitude (m.a.s.l)	Altitude	340 km^2^	WorldClim2	436	1018	581	549	3–1,895	11–2,342
Slope (°)	Slope		SRTM	4.49	4.45	3.38	4.2	0.93–26.54	0.93–26.54
Distance from the nearest water source (m)	Water_distance	1:1,000,000	FAO	1778	1890	2530	2002	0.445–15,724	1.6–8990
Human influence index	Human influence	1 km	NASA	21.2	21.3	8.74	10.8	1–52	1–52
Human footprint index	Human Footprint	1 km	NASA	15.18	15.9	8.31	9.7	1–46	1–42

### Variable importance

In general, geography, precipitation patterns, temperature variations, and environmental parameters within the region play a significant role in shaping the distribution of both *Biomphalaria* and *Bulinus*, although their relative contributions to the two models vary across the region and the method for the detection of variable importance (Fig. 1). Parameters highlighted in blue are considered strong predictors with significant influence, while those in black exhibit minor influence, and those with negative variable importance values in red are considered non-significant predictors according to the MDA metric (Fig. 1, left). We based on the same order of feature importance in MDA to categorize results in MDG into blue, black, and red (Fig. 1, right). Specifically, the most influential features affecting the distribution of *Biomphalaria* IH snails include altitude, and mainly climatic features, i.e. precipitation during the wettest month (BIO13), mean annual temperature (BIO1), and mean annual precipitation (BIO12). Features with minor contributions to the *Biomphalaria* model include the remaining climatic features like precipitation during the driest month (BIO14), temperature of the coldest month (BIO6), temperature of the warmest month (BIO5), and soil related features, as well as land cover. Additionally, the Human Footprint, land surface temperature, water distance, and vegetation index were found to have a lesser significance only with the MDA metric. The other parameters were found to be non-significant for the *Biomphalaria* model.

For *Bulinus*, the most significant parameters influencing its distribution are altitude, and again climatic features such as precipitation during the wettest month (BIO13), mean annual temperature (BIO1), mean annual precipitation (BIO12), as well as some soil features (nitrogen concentration, clay content). All other features are less relevant. Of these, parameters like land surface temperature, water distance, vegetation index, bulk density of fine earth fraction, and slope were found to be significant only when using the MDA method and only to a minor degree. Parameters that were not found to significantly impact the *Bulinus* IH species distribution at all include soil pH, organic carbon content, and the Human Footprint, amongst others (Fig. 1).

### Predicted probabilities for the occurrence of IH snails

**Figure 2:**
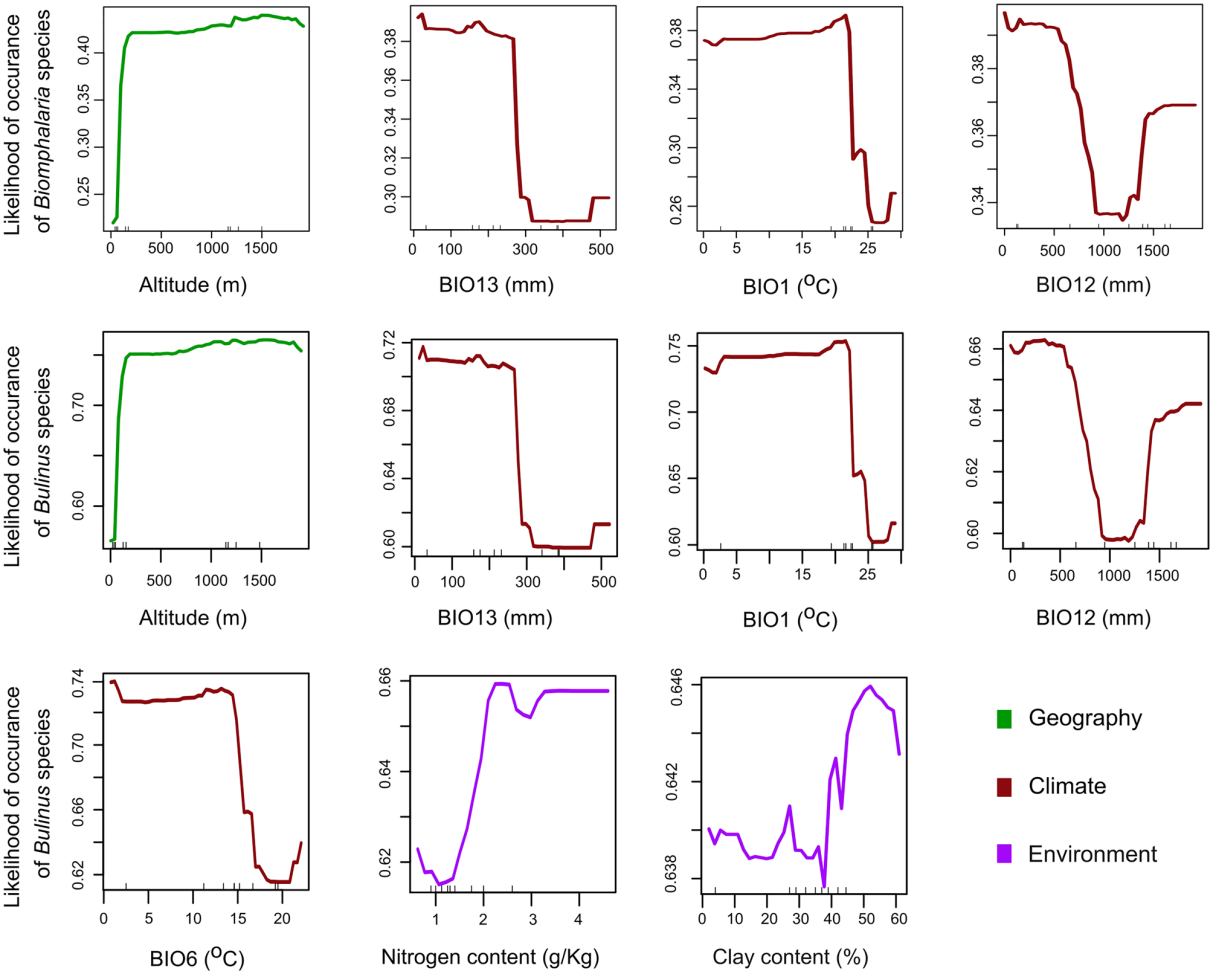
Likelihood of *Biomphalaria* species (1st panel) and *Bulinus* species occurrence (2nd and 3rd panels) in relation to the significant featuresfeatures identified by both importance metrics (MDA and MDG) in the *Biomphalaria* and *Bulinus* models. (Compare Fig. 1; see Supplementary S1 Fig. 2 for Biomphalaria and Fig. 3 for *Bulinus* predicted probabilities for the remaining predictors.

The simulated probabilities of genus occurrence in relation to the significant features identified in Fig. 1 demonstrate non-linear relationships, for both *Biomphalaria* and *Bulinus* IH snails. The likelihood of encountering *Bulinus* species is generally higher than that of *Biomphalaria* species in the region based on their probability values (Fig. 2). Nevertheless, the predicted probabilities for both genera exhibit consistent patterns concerning the importance of altitude, precipitation during the wettest month (BIO13), mean annual temperature (BIO1), and annual precipitation (BIO12). As altitude increases, the probabilities of occurrence for both IHs exhibit a steep rise up to an elevation of approximately 500 m.a.s.l. Beyond this point, the occurrence gradually increases, albeit at a very gradual rate, until approximately 1500–1800 m.a.s.l., where the trend peaks with a noticeable decrease in the likelihood of encountering these species (Fig. 2).

Conversely, for the occurrence of both IHs, the predicted probabilities decrease with a rise in precipitation levels less than 300 mm in the wettest month (BIO13) and annual precipitation (BIO12) of less than 1000 mm. This is followed by a slight increase at the end of the trend for BIO13 and a strong increase for BIO12 between around 1250 to 1900 mm. The feature mean annual temperature (BIO1) shows a complex relationship, indicating a gradual increasing trend towards higher values between 20 and 25 °C, followed by a steep probability decrease. Additionally, the association with the temperature of the coldest month (BIO6) indicates a decreasing probability of encountering *Bulinus* between 8 and 20 °C, followed by a slight increase up to 22 °C. The probability of encountering *Bulinus* increases with an increase in the soil nitrogen content, with a high probability occurring above 2 g/kg. However, the association with clay soils is complex, generally exhibiting an increasing trend that peaks at ~ 50% content of clay soils, followed by a slight decrease up to 60%.

## Discussion

In this research, we relied on geographical data sourced from literature and the GBIF database to investigate the distribution of *Biomphalaria* and *Bulinus* IH snails for *Schistosoma* within the East African region. We observed minimal variation in the potential determinants of the distribution of both *Biomphalaria* and *Bulinus* snails across the regional scale. Geography and climate played a significant role in the distribution of *Biomphalaria*, while geography, climate, and to some extent, several soil factors, were crucial factors shaping the presence of *Bulinus* snails. However, it is crucial to note that the varying significance of parameters, highlights the intricate nature of snail behavior and distribution. Numerous interacting factors can convolute the straightforward impact of specific parameters potentially attenuating their effects in the model. In the following sections, we discuss IH snail occurrence in relation to the significant, minor, and non-significant predictor features within an ecological context.

### Most significant predictor features of IH snail occurrences

The identification of significant features for both IH groups relied on high variable importance values, and similar results in both MDA and MDG metrics. Nonetheless our findings reveal that both genera thrive better below 500 m.a.s.l of altitude, potentially because lower altitudes promote stagnant water, facilitating breeding, while higher altitudes facilitate water flow^56^, a reflection of the dispersal patterns of the IH snails^14^. Thus, the variation in the altitude of the study area plays a pivotal role, although it is important to note that Abe et al.^57^ found that altitude did not significantly impact the distribution of *Bulinus* snails which they associated with the lack of altitude variation in their study area. Nonetheless, our findings complement the previous research studies which have reported differing upper altitude limits for IH snail occurrence in Uganda, with values ranging from 1400 m a.s.l^58^, to more than 1600 m a.s.l^14^, and even above 2000 m.a.s.l^50^. Notably, *Bulinus* species have been documented at exceptionally high altitudes (3997 m.a.s.l)^32^, showing favorable conditions at such altitudes. People in high-altitude populations are at risk of disease exposure, yet often receive minimal attention from health authorities and vector control programs, posing a significant concern for their health. Therefore, dedicated research is needed to establish an upper limit for both forms of schistosomiasis and assess their potential impact on host-parasite interactions and transmission of the disease. Additionally, further investigations are required to determine whether the observed and assumed shifts in altitudinal thresholds are attributable to climate change or other factors.

Furthermore, the foremost significant drivers affecting the distribution of both *Biomphalaria* and *Bulinus* snails according to our study are the climate features, temperature, and precipitation. In contrast, a locally restricted study in western Uganda^14^, assigned a lesser degree of importance to climate. This, suggests that the precise impact of climate change on IH snails and schistosomiasis is likely to exhibit variations based on geographical or spatio-temporal scales under consideration^59^. Precipitation serves as a critical metric for assessing the availability of suitable water bodies that snails are known to inhabit^36^. For example, climate change can lead to fluctuations in regional precipitation levels, which may in turn modify transmission patterns and the onset of schistosomiasis^36,38^. Nonetheless, an increase in precipitation levels contributes to the proliferation of breeding sites by increasing surface runoff into freshwater ecosystems^60^, thereby enhancing the supply of organic matter, which serves as food for the snails, ultimately promoting their growth and fecundity^60,61^. Moreover, precipitation events provide suitable conditions for snails to emerge from estivation within temporary breeding sites, coinciding with a higher peak of reproduction among these organisms^62^. This would also explain the strong increase of IH snails’ occurrence with precipitation features we found in our analysis. However, it is worth noting that excessive precipitation can also have adverse consequences on the distribution of IH snails^60^. Heavy rainfall can cause the breeding sites to be flooded, which dislocates snails and leads to a decline in snail populations. Consequently, snails disperse to new locations, establishing new areas for these vectors and posing a risk for the renewed transmission of schistosomiasis^60^. In contrast, during dry seasons, precipitation levels are low, and snails need to adapt, can undergo aestivation and their occurrence reduces, this may be a possible explanation for the negative correlation with the precipitation during the warmest months.

In a comparative context, our study emphasizes the importance of temperature in shaping snail distribution patterns across the broader East African region. Generally, freshwater snails are ectothermic, meaning their body temperature is regulated by the surrounding environment^12^. Temperature plays a crucial role in determining the development, survival, and reproductive rates of snails, as corroborated by multiple studies^10,36–38,56,63^. Interestingly, within the more confined geographical scope of Western Uganda, temperature exhibited a considerably weaker influence on the distribution of IH species^14^. This could be attributed to the more consistent temperature fluctuations compared to the broader variations seen in larger-scale studies like ours. At a broader spatial scale, our study reveals a pronounced prevalence of intermediate host snails when mean annual temperatures range between 20 and 25 °C. In prior studies, a temperature of 25 °C has been associated with an increase in snail populations^64,65^. In addition, Malone^65^ noticed an ideal temperature range of 20–27 °C for the intramolluscan development of *S. mansoni* within *Biomphalaria* spp. snails. On the other hand, decreased probability of IH snail presence during warm seasons exceeding 29 °C as shown in our study can be attributed to elevated snail mortality, diminished reproductive capacity, and inhibited snail growth, ultimately resulting in reduced schistosomiasis cases in such seasons^63,66^.

In addition, the presence of clay in the soil was a significant factor in the *Bulinus* model, consistent with prior research by Stensgaard et al.^36^, which associated clay-rich soils with higher snail prevalence. However, other studies suggested that clay content in the soil had only a minimal impact on IH snail presence^46,67^. Nonetheless, clay content in the nearby terrestrial surroundings can influence the distribution of IH snails by affecting soil texture, water retention, and drainage. The presence of heightened clay content may foster waterlogged conditions that are favorable for the proliferation of IH snails^56^. The strong relationship between soil nitrogen content and *Bulinus* IH snail distribution implies that even minor variations in soil nitrogen content can significantly impact their distribution. This connection suggests that although snails typically flourish in aquatic environments, the presence of soil nitrogen levels in the nearby terrestrial surroundings might affect the spread of *Bulinus* snails. Theoretically, increased soil nitrogen often correlates with a greater chance of nitrogen leaching, which could lead to elevated nitrogen levels in streams or floodplain habitats. These conditions could favor the survival and proliferation of these snails within their aquatic environments.

### Minor and non-significant predictor features of IH snail occurrence

Certain predictor features held relatively low importance on the distribution of both genera. Discrepancies between the MDA and MDG metrics regarding these parameters were noted. A brief discussion of possible explanations for the limited and non-significant significance of these parameters on the distribution of IH snails is provided, taking into account conflicting findings in the literature. The limited impact of some climate features like BIO14 and BIO5 during the driest month can be attributed to factors including food scarcity, snail adaptations, and the possibility of aestivation/hibernation, with the likelihood of snail mortality during these driest months^62,63,66,68^. Scenarios like hibernation often occurs as most temporary breeding sites dry out^68^. Moreover, the feeding habits of freshwater snails can be influenced by cold temperatures (BIO6), leading to a potential decrease in their reproductive activity^61^. In fact, studies typically indicate that precipitation and temperature play a minimal role or lack statistical significance in influencing the distribution of intermediate host snails^14,46^. This can be linked to the smaller geographical scope examined in prior studies, where similar climatic changes were observed, resulting in collinearity in the climate data^14,46^. Consequently, there was limited variation in the data, hindering the reflection of the significance of climate variables as primary drivers for snail distribution. In contrast, our regional and larger-scale study provides a more comprehensive perspective. However, it is important to note that the distribution of IH snails may not solely be driven by all climate features but can also be influenced by a complex interplay of various factors including ecological, topological, and human factors^12,59^.

Sand content, as observed, emerged as a significant yet a minor feature in both the *Biomphalaria* and *Bulinus* models. This finding is in line with the research conducted by Stensgaard et al.^36^, which highlights the significance of specific levels of sand content in snail distribution. Sandy soils, due to their inherent characteristics that enhance drainage, significantly impact the suitability of habitats for snails^36^. However, sand content, representing fine soil particles, may not consistently exert a strong influence on the distribution of IH snails, with its impact varying potentially based on its content for example 34–39% in our study area. On the other hand, the significance of silt content in *Bulinus* presence was notably lower, as indicated only by MDA. This finding aligns with the results reported by Deka^46^, underscoring the limited contribution of silt content to defining the presence of IH snails. On the contrary, Olkeba et al.^67^ observed higher *Bulinus globosus* populations in regions with higher silt content. However, it is crucial to acknowledge that the association between soil texture (silt, clay, sand) and snail distribution represents only one aspect within a larger ecological framework. This framework includes various factors like water chemistry, vegetation, and climate.

Furthermore, we observed that soil pH (levels 5.1–9.2), had minimal significance in the distribution of *Bulinus* snails and was not significant at all in the *Biomphalaria* model. The limited impact in our study could be attributed to the varying alkaline nature of the soils. Likewise, the restricted importance of both bulk density of the fine earth fraction and soil cation exchange capacity, as constituents of soil compositions, can be linked to the limited influence exerted by the soil content parameters (sand, silt, clay). It is essential to acknowledge that land use distribution involves various classes, which vary by region and over time^69^. The potential impact of land use on the distribution of IH snails, such as waterbodies and cropland vegetation mosaics, may be limited by superimposing effects from irrelevant factors like savannah and barren land^56^. The relatively minor impact of the human footprint, which was a weaker predictor for *Biomphalaria* snail distribution (by MDA), is in line with findings from Olkeba et al.^67^ and Krauth et al.^70^. Nonetheless, humans often play a crucial role in introducing snails into new environments and serve as passive dispersal vectors^70,71^ through expansion of irrigation agriculture, settlement and fishing activities. Conversely, a study by Tabo et al.^14^ did not identify human influence as a significant factor affecting IH snail distribution, potentially because some of the habitats are in reversed areas and in game parks where human activities are limited^14^. Furthermore, this variance may be attributed to the limited spatial scope of their case study, which may not comprehensively capture the full extent of human impact on snail distribution. While Deka^46^ emphasized the importance of proximity to the nearest water body as a significant variable, our research indicates its limited influence on the distribution of both genera. Surprisingly, Tabo et al.^14^ reached a similar conclusion regarding the insignificance of this variable in IH snail distribution. These disparities may stem from the distinctive geographical and landscape characteristics considered.

In our study, with an NDVI range of 0.18 to 0.83 and an LST range of 3.4 to 43.6 °C in the region, both parameters exhibited low significance in determining the distribution of both genera (by MDG). This observation aligns with previous studies conducted by Magero et al.^26^, Boitt and Suleiman^56^, and Deka^46^, all of which found a similar limited influence of these two parameters on the presence of IH snails. Nonetheless, it is important to consider that we have observed in this study that land cover has a limited influence at all. Moreover, Boitt and Suleiman^56^ have pointed out that land surface temperature (LST) is significantly shaped by land cover, while NDVI indirectly reflects land cover characteristics. This interrelationship may help explain the relatively modest impact of both LST and NDVI on snail distribution in our study.

While the study provides valuable new insights and results, it is limited by the scarcity of accessible physico-chemical data from online spatial databases or literature in the entire region or from major parts of the study area. The sole available physico-chemical data from a survey field study^14^ is constrained to a localized area in Western Uganda within our study region. Nevertheless, we advocate for extensive field sampling studies across East Africa.

## Conclusion

Our comprehensive analysis highlights the significance of geographical, climatic, environmental, and human factors in understanding the distribution of IH snails for schistosomiasis. Such factors can influence not only the occurrences of the genera but specifically their speciation, extinction and dispersion processes in an ecosystem. Our machine-learning approach disentangled key drivers, revealing that topography and climate predominantly influence *Biomphalaria*, while topography, climate, soil content, and nitrogen concentration collectively affect the presence of *Bulinus*. The intricate relationship with topography (altitude) may reflect dispersal limitations or environmental filtering, while positive associations with precipitation patterns and temperature variations suggest the prevalence of IH snails in East African ecosystems, especially within the tropical climate zone. Furthermore, clayish soil content and high nitrogen levels favor IH snail distribution in freshwater habitats. It is crucial to acknowledge the multifaceted nature of IH snail distribution, influenced by diverse ecological, climatic, topological, and human factors with varying contributions. These findings provide a foundational dataset for future research and risk mapping, supporting targeted prevention and control efforts against schistosomiasis. In addition, the findings have significant implications for public health. Policy makers and stakeholders should consider habitat suitability and prioritize actions on features identified as significant for the distribution of IH snails in the region. It is crucial to integrate approaches and enhance community awareness regarding these significant factors, leading to the design and implementation of integrative measures for the control of IHs and, consequently, the prevention of schistosomiasis.

### Supplementary Information

Supplementary Information.

Supplementary Table S1.

## Data Availability

The data generated or analyzed are present in the manuscript.

## References

[1] WHO, World Health Organization. Combating neglected tropical disease. https://www.un.org/africarenewal/magazine/february-2023/combating-neglected-tropical-diseases (Accesed October 2023) (2023).

[2] Aula OP, McManus DP, Jones MK, Gordon CA (2021). Schistosomiasis with a focus on Africa. Trop. Med. Infect..

[3] Hotez PJ (2014). The global burden of disease study 2010: Interpretation and implications for the neglected tropical diseases. PLoS Negl. Trop. Dis..

[4] Conteh L, Engels T, Molyneux DH (2010). Socioeconomic aspects of neglected tropical diseases. Lancet.

[5] WHO, World Health Organization. Schistosomiasis. https://www.who.int/news-room/fact-sheets/detail/schistosomiasis (Accesed October 2023) (2023).

[6] Colley DG, Bustinduy AL, Secor WE, King CH (2014). Human schistosomiasis. Lancet.

[7] Utzinger J (2009). Schistosomiasis and neglected tropical diseases: Towards integrated and sustainable control and a word of caution. J. Parasitol..

[8] Fenwick A, Jourdan P (2016). Schistosomiasis elimination by 2020 or 2030?. Int. J. Parasitol..

[9] Kittur N (2019). Persistent hotspots in schistosomiasis consortium for operational research and evaluation studies for gaining and sustaining control of schistosomiasis after four years of mass drug administration of praziquantel. Am. J. Trop. Med. Hyg..

[10] Díaz AV, Walker M, Webster JP (2023). Reaching the World Health Organization elimination targets for schistosomiasis: The importance of a one health perspective. Philos. Trans. R. Soc..

[11] World Health Organization. Ending the neglect to attain the sustainable development goals: A road map for neglected tropical diseases 2021–2030. https://www.who.int/publications/i/item/9789240010352 (2020).

[12] Douchet P, Gourbal B, Loker ES, Rey O (2023). Schistosoma transmission: Scaling-up competence from hosts to ecosystems. Trends Parasitol..

[13] Sokolow SH (2016). Global assessment of schistosomiasis control over the past century shows targeting the snail intermediate host works best. PLoS Negl. Trop. Dis..

[14] Tabo Z (2022). Factors controlling the distribution of intermediate host snails of Schistosoma in Crater Lakes in Uganda: A machine learning approach. Front. Environ. Sci..

[15] Bakuza JS (2017). Assessing *S*. *mansoni* prevalence in Biomphalaria snails in the Gombe ecosystem of western Tanzania: The importance of DNA sequence data for clarifying species identification. Parasit. Vectors.

[16] Breiman L (2001). Random forests. Mach. Learn..

[17] Huang BFF, Boutros PC (2016). The parameter sensitivity of random forests. BMC Bioinform..

[18] Schonlau M, Zou RY (2020). The random forest algorithm for statistical learning. Stata J..

[19] Collin FD (2021). Extending approximate Bayesian computation with supervised machine learning to infer demographic history from genetic polymorphisms using DIYABC random forest. Mol. Ecol. Resour..

[20] Georganos S (2021). Geographical random forests: A spatial extension of the random forest algorithm to address spatial heterogeneity in remote sensing and population modelling. Geocarto Int..

[21] Boulesteix A-L, Janitza S, Kruppa J, König IR (2012). Overview of random forest methodology and practical guidance with emphasis on computational biology and bioinformatics. WIREs Data Min. Knowl. Discov..

[22] Bunyamin H, Tunys T (2016). A comparison of retweet prediction approaches: The superiority of random forest learning method. Telkomnika.

[23] Zhang J (2020). Risk prediction of two types of potential snail habitats in Anhui Province of China: Model-based approaches. PLoS Negl. Trop. Dis..

[24] Boonprong S, Cao C, Chen W, Bao S (2018). Random forest variable importance spectral indices scheme for burnt forest recovery monitoring-multilevel RF-VIMP. J. Remote Sens..

[25] Brieuc MSO, Waters CD, Drinan DP, Naish KA (2018). A practical introduction to random forest for genetic association studies in ecology and evolution. Mol. Ecol. Resour..

[26] Magero VO, Kisara S, Wade CM (2021). Geographical distribution of *Biomphalaria* snails in East Africa. bioRxiv.

[27] Worldometer: Eastern Africa Population. https://www.worldometers.info/world-population/eastern-africa-population/ (2023).

[28] Salzburger W, Van Bocxlaer B, Cohen AS (2014). Ecology and evolution of the African Great Lakes and their faunas. Annu. Rev. Ecol. Evol. Syst..

[29] Spigel RH, Coulter GW, Whittaker KT, Johnson TC, Odada EO (2019). Comparison of hydrology and physical limnology of the East African great lakes: Tanganyika, Malawi, Victoria, Kivu and Turkana (with reference to some North American Great Lakes). Limnology, Climatology and Paleoclimatology of the East African lakes.

[30] Gryseels B, Polman K, Clerinx J, Kestens L (2006). Human schistosomiasis. Lancet.

[31] Chibwana FD, Tumwebaze I, Mahulu A, Sands AF, Albrecht C (2020). Assessing the diversity and distribution of potential intermediate hosts snails for urogenital schistosomiasis: *Bulinus* spp. (Gastropoda: Planorbidae) of Lake Victoria. Parasit. Vectors.

[32] Tumwebaze I, Clewing C, Chibwana FD, Kipyegon JK, Albrecht C (2022). Evolution and biogeography of freshwater snails of the genus *Bulinus* (Gastropoda) in afromontane extreme environments. Front. Environ. Sci..

[33] GBIF.org (22 May 2023) GBIF Occurrence Download. 10.15468/dl.6esfpk (2023).

[34] R Core Team R: A Language and Environment for Statistical Computing. Version 4.0.3. R Foundation for Statistical Computing. http://www.R-project.org (2020).

[35] Gorelick N (2017). Google earth engine: Planetary-scale geospatial analysis for everyone. Remote Sens. Environ..

[36] Stensgaard AS (2013). Large-scale determinants of intestinal schistosomiasis and intermediate host snail distribution across Africa: Does climate matter?. Acta Trop..

[37] McCreesh N, Nikulin G, Booth M (2015). Predicting the effects of climate change on *Schistosoma*
*mansoni* transmission in eastern Africa. Parasit. Vectors.

[38] McCreesh N, Arinaitwe M, Arineitwe W, Tukahebwa EM, Booth M (2014). Effect of water temperature and population density on the population dynamics of *Schistosoma Mansoni* intermediate host snails. Parasit. Vectors.

[39] Fick SE, Hijmans RJ (2017). WorldClim 2: New 1-km spatial resolution climate surfaces for global land areas. Int. J. Climatol.

[40] Wan, Z., Hook, S., Hulley, G. (2021). *MODIS/Terra Land Surface Temperature/Emissivity Daily L3 Global 1km SIN Grid V061* [Data set]. NASA EOSDIS Land Processes DAAC. 10.5067/MODIS/MOD11A1.061 (Accessed October 2023) (2023).

[41] Didan, K. *MODIS/Terra Vegetation Indices 16-Day L3 Global 250m SIN Grid V061*. NASA EOSDIS Land Processes DAAC. Accessed 2023-06-13 from 10.5067/MODIS/MOD13Q1.061 (Accessed October 2023) (2021).

[42] Pettorelli N (2014). Satellite remote sensing for applied ecologists: Opportunities and challenges. J. Appl. Ecol..

[43] Turner W (2015). Free and open-access satellite data are key to biodiversity conservation. Biol. Conserv..

[44] Oso OG, Odaibo AB (2021). Land use/land cover change, physico-chemical parameters and freshwater snails in Yewa North, Southwestern Nigeria. PLoS One.

[45] Friedl, M., Sulla-Menashe, D. *MODIS/Terra+Aqua Land Cover Type Yearly L3 Global 500m SIN Grid V061*. NASA EOSDIS Land Processes DAAC. 10.5067/MODIS/MCD12Q1.061 (Accessed October 2023) (2022).

[46] Deka MA (2022). Predictive risk mapping of Schistosomiasis in Madagascar using ecological Niche modeling and precision mapping. Trop. Med. Infect..

[47] Hengl T (2021). African soil properties and nutrients mapped at 30 m spatial resolution using two-scale ensemble machine learning. Sci. Rep..

[48] Batjes NH, Ribeiro E, Van Oostrum A (2020). Standardised soil profile data to support global mapping and modelling (WoSIS snapshot 2019). Earth Syst. Sci. Data.

[49] Hauffe T, Albrecht C, Wilke T (2016). Assembly processes of gastropod community change with horizontal and vertical zonation in ancient Lake Ohrid: A metacommunity speciation perspective. Biogeosciences.

[50] Stanton MC (2017). Intestinal schistosomiasis in Uganda at high altitude (> 1400 m): Malacological and epidemiological surveys on Mount Elgon and in Fort Portal crater lakes reveal extra preventive chemotherapy needs. Infect. Dis. Poverty.

[51] Farr TG (2007). The shuttle radar topography mission. Rev. Geophys..

[52] Jenness, J., Dooley, J., & Riva, C. African Water Resource Database: GIS-based tools for inland aquatic resource management: 1. Concepts and application case studies 33 (CIFA technical paper, 2007).

[53] Wildlife Conservation Society - WCS, and Center for International Earth Science Information Network - CIESIN - Columbia University. Last of the Wild Project, Version 2, 2005 (LWP-2): Global Human Influence Index (HII) Dataset (Geographic). NASA Socioeconomic Data and Applications Center (SEDAC) (2005).

[54] Berrar D, Ranganathan S, Gribskov M, Nakai K, Schönbach C (2019). Cross-validation. Encyclopedia of Bioinformatics and Computational Biology.

[55] Evans JS, Murphy MA, Holden ZA, Cushman SA, Drew CA, Wiersma YF, Huettmann F (2011). Modeling species distribution and change using random forest. Predictive Species and Habitat Modeling in Landscape Ecology.

[56] Boitt MK, Suleiman MK (2021). Mapping of freshwater snails’ habitat—A source of transmitting Bilharzia in Mwea sub-county, Kenya. J. Geosci. Environ. Prot..

[57] Abe EM (2012). Predicting the geospatial distribution of *Bulinus* snail vector of urinary schistosomiasis in Abeokuta, South-Western, Nigeria. Zool.

[58] Kabatereine NB, Brooker S, Tukahebwa EM, Kazibwe F, Onapa AW (2004). Epidemiology and geography of *Schistosoma mansoni* in Uganda: Implications for planning control. Trop. Med. Int. Health.

[59] Stensgaard AS, Vounatsou P, Sengupta ME, Utzinger J (2019). Schistosomes, snails and climate change: Current trends and future expectations. Acta Trop..

[60] David NF (2018). Spatial distribution and seasonality of biomphalaria spp. In São Luís (Maranhão, Brazil). Parasitol. Res..

[61] Madsen H, Coulibaly G, Furu P (1987). Distribution of freshwater snails in the river Niger basin in Mali with special reference to the intermediate hosts of schistosomes. Hydrobiologia.

[62] Brooker S (2002). Use of remote sensing and a geographical information system in a national helminth control programme in Chad. Bull. World Health Organ..

[63] Tabo Z, Kalinda C, Breuer L, Albrecht C (2023). Adapting strategies for effective schistosomiasis prevention: A mathematical modeling approach. Mathematics.

[64] Manyangadze T, Chimbari MJ, Gebreslasie M, Ceccato P, Mukaratirwa S (2016). Modelling the spatial and seasonal distribution of suitable habitats of schistosomiasis intermediate host snails using Maxent in Ndumo area, KwaZulu-Natal Province, South Africa. Parasit. Vectors.

[65] Malone JB (2005). Biology-based mapping of vector-borne parasites by geographic information systems and remote sensing. Parassitologia.

[66] Kalinda C, Chimbari M, Mukaratirwa S (2017). Implications of changing temperatures on the growth, fecundity and survival of intermediate host snails of schistosomiasis: A systematic review. Int. J. Environ. Res. Public Health.

[67] Olkeba BK (2020). Environmental and biotic factors affecting freshwater snail intermediate hosts in the Ethiopian Rift Valley region. Parasit. Vectors.

[68] Perez-Saez J (2016). Hydrology and density feedbacks control the ecology of intermediate hosts of schistosomiasis across habitats in seasonal climates. Proc. Natl. Acad. Sci..

[69] Lambin EF, Geist H, Rindfuss RR (2005). Land-use and land-cover change: Developing and implementing an agenda for local processes with global impacts. IHDP Update.

[70] Krauth SJ (2017). Distribution of intermediate host snails of schistosomiasis and fascioliasis in relation to environmental factors during the dry season in the Tchologo region, Côte d’Ivoire. Adv. Water Resour..

[71] Kappes H, Haase P (2012). Slow, but steady: Dispersal of freshwater, molluscs. Aquat. Sci..

