# Supplementary material for: A machine learning approach for modeling the occurrence of the major intermediate hosts for schistosomiasis in East Africa
Source: Sci Rep. 2024 Feb 21;14:4274. doi: 10.1038/s41598-024-54699-1 (PMC10881506; doi:10.1038/s41598-024-54699-1)
Supplement: Supplementary file 1 — Supplementary Information. [file 41598_2024_54699_MOESM1_ESM.docx]

**Supplementary file S1 for;**

**A machine learning approach for modeling the occurrence of the major intermediate hosts for schistosomiasis in East Africa**

**Zadoki Tabo^1,2*^, Lutz Breuer^2,3^, Codalli Fabia^2^, Gorata Samuel^4^ & Christian Albrecht^1^**


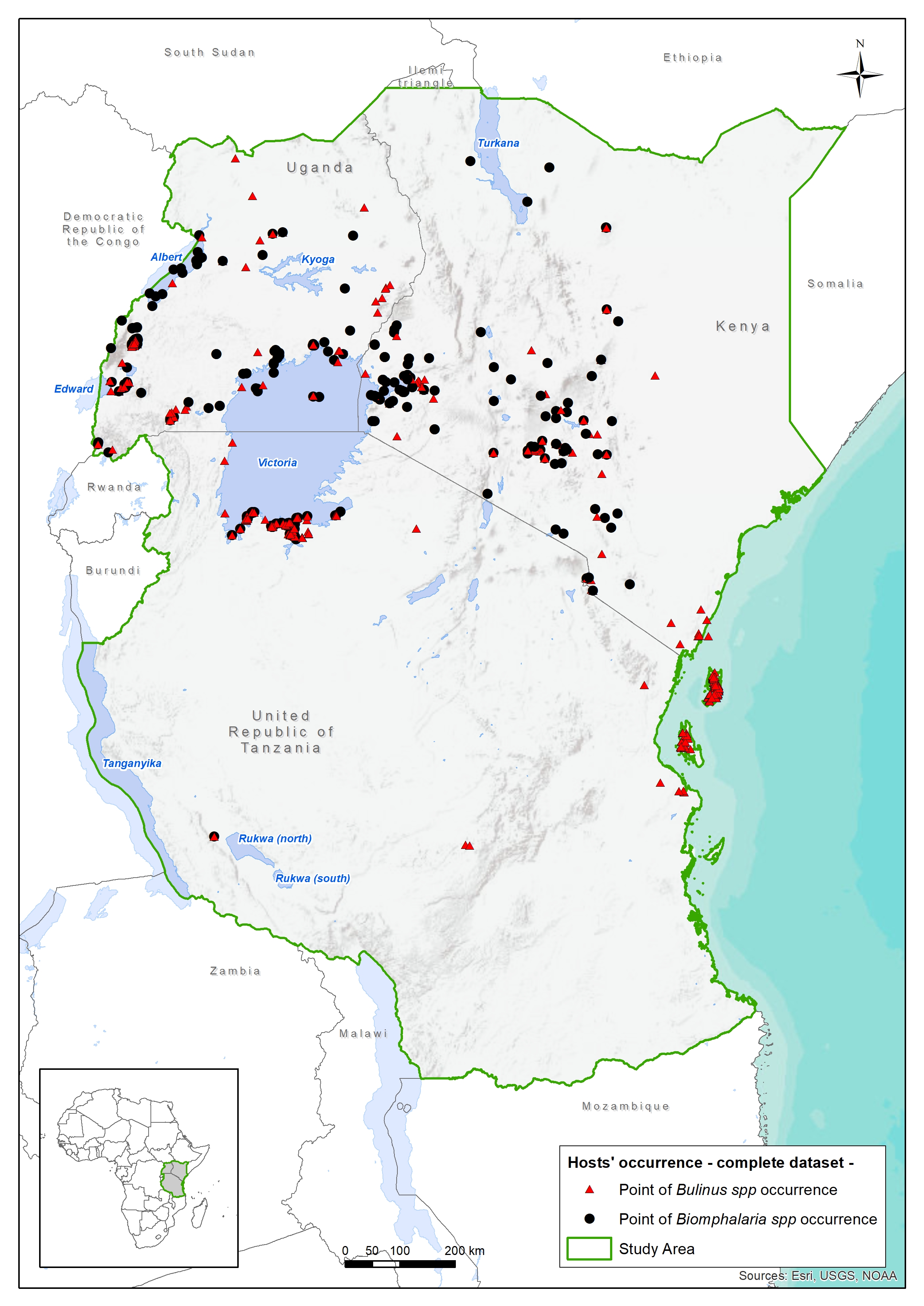


**Figure1**: Geographic distribution of occurrence data for *Biomphalaria* spp. (black) and *Bulinus* spp. (red) in the study area of East Africa. For *Bulinus* spp., only species known to be actual intermediate hosts are considered (see text for details). The map was generated using the software ArcGIS desktop (ESRI 2019. ArcGIS Desktop: Release 10. Redlands, CA: Environmental Systems Research Institute; <https://desktop.arcgis.com/en/arcmap/latest/get-started/introduction/whats-new-in-arcgis.htm>)


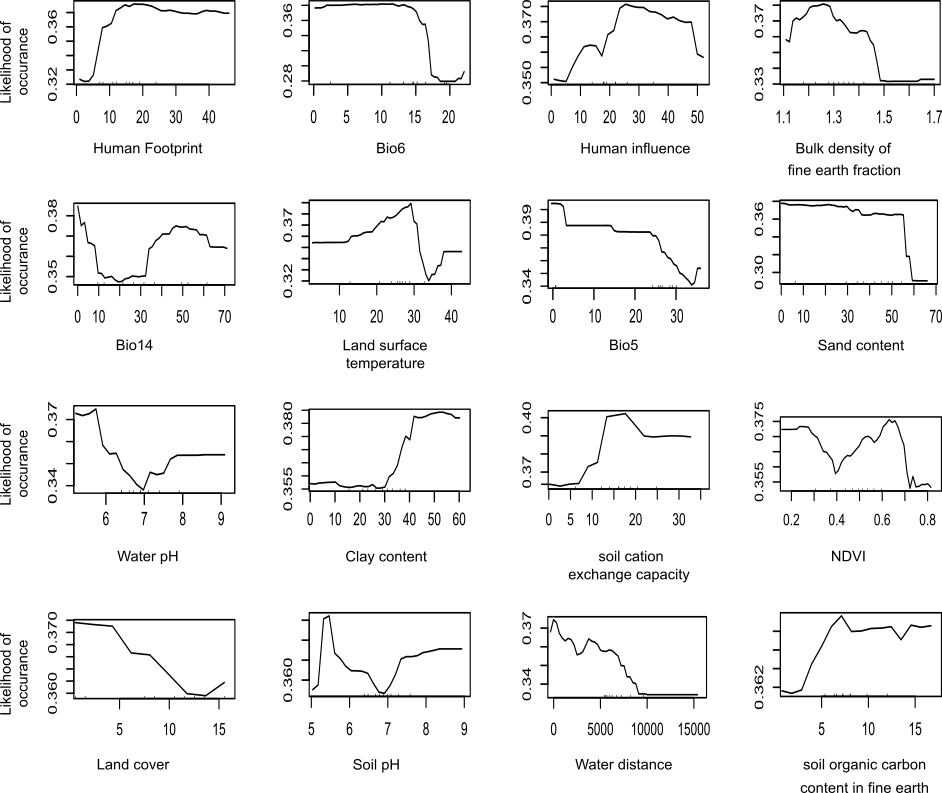


**Figure 2**. Likelihood of *Biomphalaria* species occurrence in relation to the weak and non-significate variables predictor variables


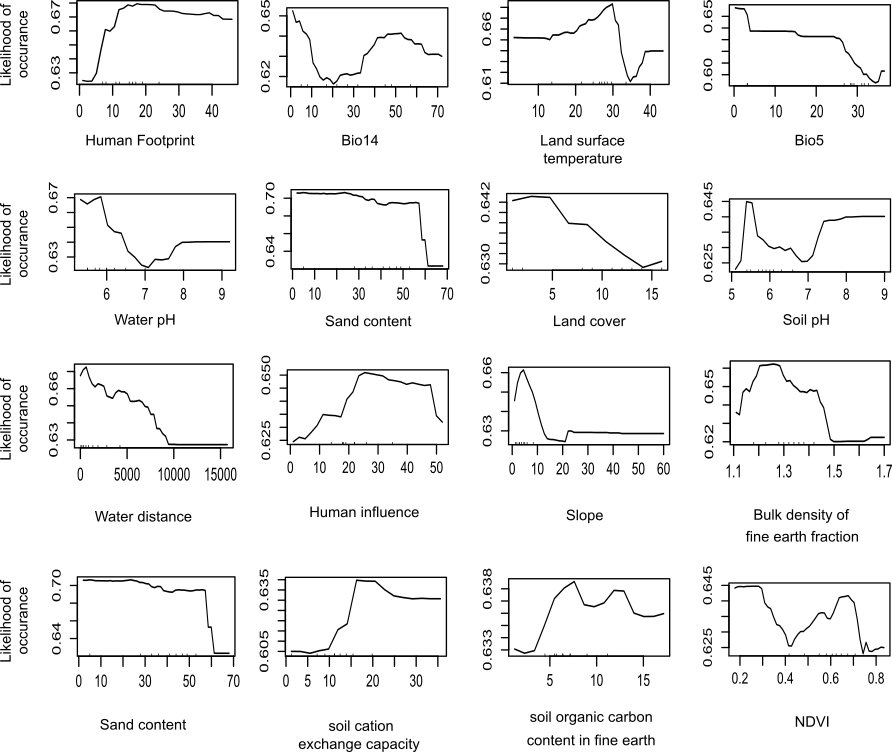


**Figure 3**. Likelihood of *Bulinus* species occurrence in relation to the weak and non-significate variables predictor variables

**Table S1**. Genus and species classification of *Biomphalaria* and *Bulinus* snails, along with species occurrence data (latitude and longitude), data sources and presence/absence information, including associated predictor variables.

This Table presents predictor variables that were assessed for their importance in influencing the distribution of intermediate host snails. They include geographical and topographic features (altitude, slope, distance to nearest waterbody), climatic features (mean annual temperature, BIO1, Temperature of the warmest month, BIO5, Temperature of the coldest month, BIO6, mean annual precipitation, BIO12, precipitation of the wettest month, BIO 14, precipitation of the driest month, BIO14, land surface temperature), environmental features (soil pH, water pH, Nitrogen content, sand, silt and clay content, Normalized vegetation index, Bulk density of fine earth fraction, soil cation exchange capacity, soil organic carbon content in fine earth), and Human factors (human influence index and Footprint index)
